# Navigating the Host Cell Response during Entry into Sites of Latent Cytomegalovirus Infection

**DOI:** 10.3390/pathogens7010030

**Published:** 2018-03-16

**Authors:** Matthew J. Murray, Nicholas E. Peters, Matthew B. Reeves

**Affiliations:** Institute of Immunity & Transplantation, University College London, Royal Free Campus, London NW3 2PF, UK; matthew.murray@ucl.ac.uk (M.J.M.); npeters1@nhs.net (N.E.P.)

**Keywords:** cytomegalovirus, cell death, innate immunity, latency

## Abstract

The host cell represents a hostile environment that viruses must counter in order to establish infection. Human cytomegalovirus (HCMV) is no different and encodes a multitude of functions aimed at disabling, re-directing or hijacking cellular functions to promulgate infection. However, during the very early stages of infection the virus relies on the outcome of interactions between virion components, cell surface receptors and host signalling pathways to promote an environment that supports infection. In the context of latent infection—where the virus establishes an infection in an absence of many gene products specific for lytic infection—these initial interactions are crucial events. In this review, we will discuss key host responses triggered by viral infection and how, in turn, the virus ameliorates the impact on the establishment of non-lytic infections of cells. We will focus on strategies to evade intrinsic antiviral and innate immune responses and consider their impact on viral infection. Finally, we will consider the hypothesis that the very early events upon viral infection are important for dictating the outcome of infection and consider the possibility that events that occur during entry into non-permissive cells are unique and thus contribute to the establishment of latency.

## 1. Introduction

The host response to infection is multi-faceted and is a concert of cell intrinsic, innate and adaptive immune responses. In higher-order eukaryotes, the adaptive response is orchestrated by specific immune cell types and plays an important role in the resolution of infection. However, all nucleated eukaryotic cells can respond to pathogen infection via the induction of cell intrinsic and innate immune responses, which represent the first line of defence upon infection [1].

One of the first host cell ‘trip wires’ is the detection of the pathogen during the initial contact and entry of the pathogen into the cell. Cells express a number of pattern recognition receptors (PRRs) [2] that detect foreign pathogen-associated molecular patterns (PAMPs) with the potential to trigger profound innate immune responses [3]. Additionally, events activated at these early stages can trigger cell suicide pathways, further contributing to protection of the host via elimination of the infected cell [4]. The central importance of these events is highlighted by the armoury of functions encoded by pathogens aimed at neutralising these responses.

In this review, we will discuss the interaction of human cytomegalovirus (HCMV) with these functions and the multiple strategies encoded by HCMV to subvert them. We will particularly focus on the subversion of these responses in the context of the establishment of lifelong latent infections and explore the alternative mechanisms of evasion employed by HCMV under conditions where a number of virally encoded inhibitors of the antiviral response are not expressed and the downstream consequences of this.

### 1.1. Clinical Manifestations of Human Cytomegalovirus

HCMV is a herpesvirus in the subfamily betaherpesvirinae. It is a ubiquitous infection, with seroprevalence approaching 100% in some populations [5], although in the developed world 40–60% of individuals will be infected by adulthood [6]. Primary infection of healthy individuals is usually asymptomatic, which is likely due to an effective immune response that controls the replication of the virus, and results in the establishment of lifelong latent infections of the host [7]. However, pathology does occur in particular patient groups that have impaired or immature immune responses. For example, HCMV infection pre-HAART was a major cause of retinitis in late-stage AIDS patients [8]. Similarly, the immune suppression required for transplantation exposes both solid organ and haemopoietic stem cell transplant recipients to HCMV-induced morbidity [9,10]. Finally, HCMV represents the primary infectious cause of disease, following congenital infection [11]. Indeed, congenital infection with HCMV is the most common cause of congenital deafness and can lead to further developmental defects such as microcephaly or intellectual disability [12,13,14]. The threat of HCMV is exacerbated by the fact that individuals are potentially at risk from primary infection (and re-infection) as well as the reactivation of their endogenous latent virus. This threat is no better observed than in the bone marrow transplant population, where it is the reactivation of the recipient’s latent virus that provides the major source of viraemia in these individuals [15]. All told, the disease burden of HCMV led it to being designated as a highest-priority pathogen in urgent need of a vaccine strategy [16].

Despite the need for a vaccine, HCMV can be treated. Ganciclovir (GCV) and derivatives, along with foscarnet and cidofovir, are potent inhibitors of viral replication [17]. However, their use is tempered by the associated side effects—primarily haematopoietic toxicity, which limits the potential in neonates and precludes use in pregnant women [18,19]. Importantly, the new HCMV antiviral letermovir [20] has been approved by the U.S. Food & Drug Administration, thus expanding the options available for clinical management. However, the issue of the lifelong latent infection remains. Whilst these drugs are effective against the lytic replication cycle of HCMV, they will have no impact against latent virus—an issue similarly important for the eradication of other persistent infections such as HIV [21,22,23]. Key to the eradication of HCMV is the development of an effective vaccine that prevents transmission or even the development of agents that may be able to selectively kill cells in which HCMV has become latent using chemical or immunological approaches [24,25,26].

### 1.2. HCMV Latency

A major site of HCMV latency is the CD34+ haematopoietic progenitor cell population resident in the bone marrow [27,28]. These progenitor cells give rise to all mature cell types in the blood. However, studies have revealed that the myelo-monoytic and dendritic cell lineages appear to be important vehicles for the carriage and reactivation of HCMV in vivo [29,30,31,32,33,34]. The mechanisms that underpin carriage are not fully understood. Originally considered a quiescent infection, latency is now argued to represent a spectrum of transcriptional activity that has been implicated in its regulation [35,36,37,38,39]. Crucially, full lytic gene expression is absent and no new progeny are made. The viral genome persists episomally and is usually present as multiple copies (6–12) within the latently infected cell [40,41]. These genomes are associated with cellular chromatin—a paradigm that is true of all herpes virus infections [42,43,44]. It is hypothesised that chromatinisation renders the viral genome capable of being regulated by host functions controlling eukaryotic gene expression. Indeed, the post-translational modification of histones at multiple viral promoters is entirely consistent with their expression during latency and reactivation [45]. It is this dependence on chromatin that underpins strategies to eliminate the latent virus using histone deacetylase inhibitors [24].

What is becoming increasingly clear is that the events that occur during the very early stages of viral infection of non-permissive cells (i.e., cells that potentially support latent infections) have an important role during the establishment of latency. For example, a failure of the virus to antagonise key antiviral structures has been implicated as a mechanism to drive latency by default due to a repression of lytic gene expression [46]. Furthermore, the activity of viral gene products expressed during latency are likely important through modulation of cell signalling pathway activity or chromatin-modifying enzymes [47,48,49,50]. What is less clear is whether HCMV benefits from a unique entry pathway to re-programme cells that are targets of latent infection? Are specific receptors utilised to promote an environment that drives latency, or is latency simply a failure to neutralise important antiviral host functions?

## 2. HCMV Entry

### 2.1. HCMV Entry into Permissive Cells

The initial entry events during the infection of cells that can support lytic replication has been extensively studied for many years, often taking advantage of knowledge derived from studies of other herpesviruses such as herpes simplex virus.

**Figure 1:**
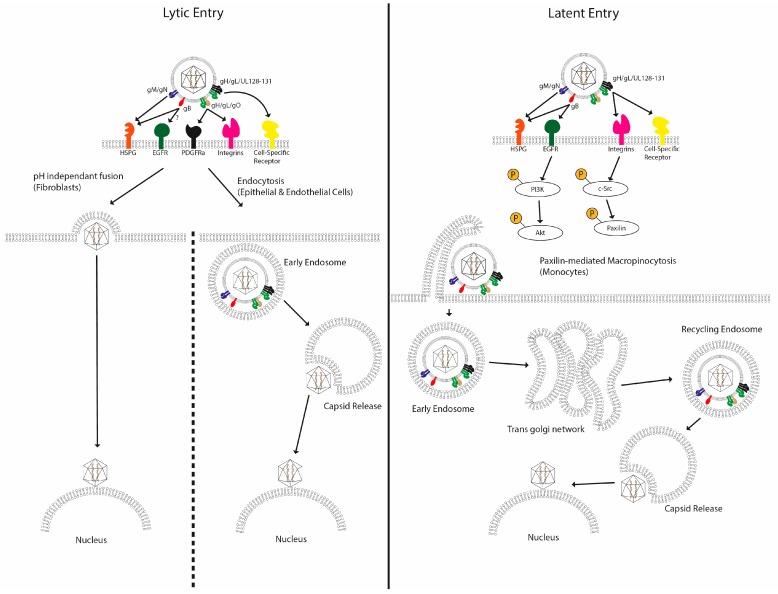
Multiple mechanisms of entry have been described for human cytomegalovirus. After initial attachment of HCMV to the plasma membrane via interactions with HSPGs, a number of high-affinity interactions have been proposed between viral glycoproteins and plasma membrane receptors in a cell-type-specific manner. These trigger specific signalling pathways, leading to viral entry though various different routes. In fibroblasts, the viral membrane fuses with the cellular membrane, releasing the contents directly into the cytoplasm. In epithelial cells, the virus is internalized via endocytosis, and in CD14+ monocytes, virus entry is proposed to occur via macropinocytosis, with the virus trafficking to the nucleus along an extended route via the trans Golgi network and recycling endosomes.

A wealth of studies has demonstrated that entry of HCMV into permissive cells is coordinated by a number of virally-encoded glycoproteins that interact with specific receptors on the cell surface—however, it is the identity of these receptors that has remained contentious (Figure 1). The classical model proposed describes the initial weak binding of the virus to the cell surface via heparan sulfate proteoglycans [51]—an event mediated by glycoproteins gB and the gM/gN complex [52]. In fibroblasts, this is followed by interactions between gB and the gH/gL/gO trimer with cellular integrins [53] and receptor tyrosine kinases (RTKs) [54]. Initial studies implicated the epidermal growth factor receptor (EGFR) as a receptor for HCMV entry into fibroblasts in 2003 [55], with gB shown to bind directly to EGFR, leading to downstream signalling events that were required for efficient HCMV infection. However, papers that attempted to recapitulate the noted ability of anti-EGFR antibodies and chemical inhibitors of EGFR signalling such as AG1478 to inhibit HCMV infection failed to do so, causing us to question the role of this RTK as a bona fide entry receptor [56]. Indeed, a subsequent study of glioblastomas revealed that the platelet-derived growth factor receptor A (PDGFRa) RTK was an important mediator of infection. Indeed, it is this RTK that has gained more traction as an important mediator of entry, with growing evidence of the importance of gH/gL/gO trimer interactions with PDGFRa during infection of fibroblasts [57,58,59], which is then followed by recruitment of gB. This is argued to be followed by pH-independent fusion between viral and cellular membranes, driven primarily by gB [60]. Consistent with gB being important in the process of fusion is the rescue of infection of a gB null virus with polyethylene glycol [61]. This model, based mainly on fibroblast infections, was complicated by studies in other cell types. The identification of an additional glycoprotein complex on the virion surface, the pentameric gH/gL/UL128-131 complex, which had mutated in the highly lab-adapted strains initially used to characterise HCMV entry, complicated the picture. The pentamer has been shown to be required for productive entry into many physiologically important cell types [62], including non-permissive CD14+ cells [63]. Additionally, different entry processes have been described in cell types other than fibroblasts, such as endocytosis in epithelial cells [64] and paxillin-dependent macropinocytosis in CD14+ cells [65]. Even the entry mechanism of HCMV into fibroblasts has been challenged, with macropinocytosis suggested to be responsible instead of pH-independent fusion [66]. All told, it appears that HCMV can exploit multiple entry pathways that may underpin the broad cellular tropism it possesses.

### 2.2. HCMV Entry into Non-Permissive Cells

The relative paucity of knowledge on the entry of HCMV into sites of latency is no doubt due in part to the relative difficulty of working with the primary cell types, in which HCMV establishes latent infections in vivo, which are CD34+ myeloid progenitor cells [27,28], compared to studies of lytic infection that tend to utilise primary fibroblasts or epithelial cells. Furthermore, it was likely assumed that the mechanism of entry into latency mirrored that observed in the more tractable permissive cell type models.

However, work mainly from the Yurochko laboratory provides the first hint of increased complexity regarding the infection of a subset of non-permissive cells (Figure 1). Circulating CD14+ monocytes are commonly used as a model of latent infection in vitro [67,68,69], with HCMV establishing a quiescent infection in them that is capable of reactivating in response to differentiation and stimulation of the cells. Given their short lifespans in vivo [70], direct infection of these cells renders them unlikely to be long-term reservoirs for the establishment of a latent HCMV in the course of a natural infection, but may aid virus dissemination around the host. However, infection of these cells can provide insight into the events that occur during the early stages of a non-permissive/latent infection. Thus, alongside CD34+ and cell lines such as THP-1 [71] (acute monocytic leukaemia), NTERA-2 [72] (embryonal carcinoma) and Kasumi-3 [73] (acute myeloblastic leukaemia), they have been utilised as useful models of latent (or at least non-permissive) HCMV infections. These in vitro models are by no means perfect [74], but they do provide useful insights and starting points for interrogation of certain aspects of HCMV latency.

Our own interests lie in the mechanisms HCMV utilises to establish latent infections of CD34+ progenitor cells. Successful infection of both CD14+ and CD34+ cells has been linked to interactions between the virion and EGFR. EGFR signalling leads to the activation of a vast array of signalling pathways including ERK/MAPK and PI3K signalling. However, when it comes to infection of sites of latency, great attention has been given to the possible role of EGFR during this process, particularly in monocytes.

Firstly, it was demonstrated that primary monocytes do actually express EGFR [75], as it was previously thought not to be expressed in cells of the haematopoetic lineage. However, those prior studies [76] were carried out using PBMCs, only a small fraction of which (5–10%) are CD14+ monocytes, which may explain the failure to detect EGFR on CD14+ cells previously. Following infection with HCMV, EGFR becomes rapidly phosphorylated in monocytes, followed by downstream phosphorylation of Akt [75]. This initiation of EGFR signalling upregulates the cytoskeletal regulator protein N-WASP, which, in concert with paxillin, is upregulated as a result of integrin signalling, not only driving the effective internalisation and trafficking of the virus, but also increasing the motility of the infected cell, which may aid the dissemination of the virus in vivo. More recently, EGFR signalling has been shown to improve the efficiency of infection of CD34+ cells as well, with EGFR-blocking antibodies and inhibitors of EGFR/PI3K signalling leading to less internalisation of virus particles if applied prior to viral infection [77]. If EGFR signalling is pharmacologically inhibited 4 h post-infection, at which point the virus is beginning to de-envelope and reach the nucleus, the establishment of latency appears to be less efficient, as more lytic (IE1/IE2) and less latent (UL138) transcripts can be detected 24 h post-infection. It is worth noting that HCMV appears to have a complex relationship with the EGFR signalling pathway during latency and reactivation. Whilst EGFR/PI3K signalling appears to play a role in entry, it is also thought to play a role in the control of viral reactivation [50]. Here the activation of EGFR is impacted by viral gene products that regulate the availability of this signalling pathway. Thus the virus activates a pathway to promote entry of CD34+ cells but then must express viral gene products to inactivate EGFR signalling post-entry to establish a latent infection [50]. Consequently, EGFR may not represent a bona fide entry receptor whose presence is absolutely required for cell entry, but engagement could play an important role in tuning the cellular environment towards one more conducive to successful viral infection.

In lytic infection of fibroblasts and epithelial cells, HCMV DNA can be detected within the nucleus approximately 30 min post-infection [78]. However, the same study suggested that HCMV takes an extended route in monocytes, requiring approximately three days for its DNA to be delivered to the nucleus. HCMV is hypothesised to be taken up into the monocyte by macropinocytosis, during which interactions between the pentameric complex and cellular integrins trigger c-Src signalling. The virus then translocates to the trans-Golgi network before moving to recycling endosomes. HCMV can seemingly be retained in the recycling endosome for multiple days before de-envelopment finally occurs, the capsid is released into the cytoplasm and it traffics to the nucleus. However, in the absence of c-Src signalling (i.e., during infection with pentamer-deficient virus), the virus is directed to the late endosome, followed by degradation. Whilst trafficking within CD34+ cells has not been subjected to a great deal of study, as stated above, DNA translocation to the nucleus takes around 4–8 h, thus occurring much more quickly than in monocytes, but slower than in fibroblasts [77]. Why the entry process is so protracted in CD14+ monocytes is intriguing and the benefits to either host or virus are unclear. Possibly a circuitous entry pathway may allow the virus to avoid certain host detection systems particularly active in these cells that would be triggered by the relatively quicker process of entry observed in many cells.

Alternatively, this extended entry pathway may be an important determinant of successful quiescent infection of monocytes. Pre-treatment of monocytes with LPS rendered them transiently permissive for IE expression 24 h following infection [79]. However, LPS did not trigger reactivation of HCMV from latently infected monocytes, nor did it promote the terminal differentiation of the monocytes to a cell type known to be permissive for HCMV lytic infection. The infection was abortive under these conditions—with no evidence of DNA replication or infectious virus production. This LPS-driven effect on HCMV could be inhibited by COX-2 inhibitor indomethacin. Intriguingly, LPS has been demonstrated to activate a COX-2-sensitive endocytic pathway in monocytes, which the virus, under these conditions, may be utilising. However, this entry pathway is not productive for the virus, as it leads neither to the production of infectious progeny nor to effective establishment of latency. Indeed IE-expressing monocytes are likely to be identified and destroyed by the immune system in vivo. Consequently, the use of this pathway may be of no intrinsic benefit to the virus in these cells but, instead, may support a model whereby the normal route of entry into the cell has important consequences downstream and thus is beneficial to the virus.

What these studies of entry into latent and lytic infection may reveal is that the virus can utilise multiple entry pathways. This begs the question: could this be important? Does the presence of different entry pathways allow the virus to tailor entry to navigate different host cell responses the virus may encounter in a cell-type-specific manner?

## 3. Host Defences in Response to HCMV Infection

Nucleated eukaryotic cells encode a number of defence mechanisms to protect them from pathogens. Central to this is the activity of cell autonomous/innate immune responses. The innate immune response to HCMV is of critical importance to the intracellular environment encountered by the virus and the routes of entry determine some of the ways the virus is detected. Furthermore, given the variety of entry routes utilised by HCMV to infect different cell types, the innate immune response may be shaped differently in different cell types.

### 3.1. Evasion of Host Cell Apoptosis

**Figure 2:**
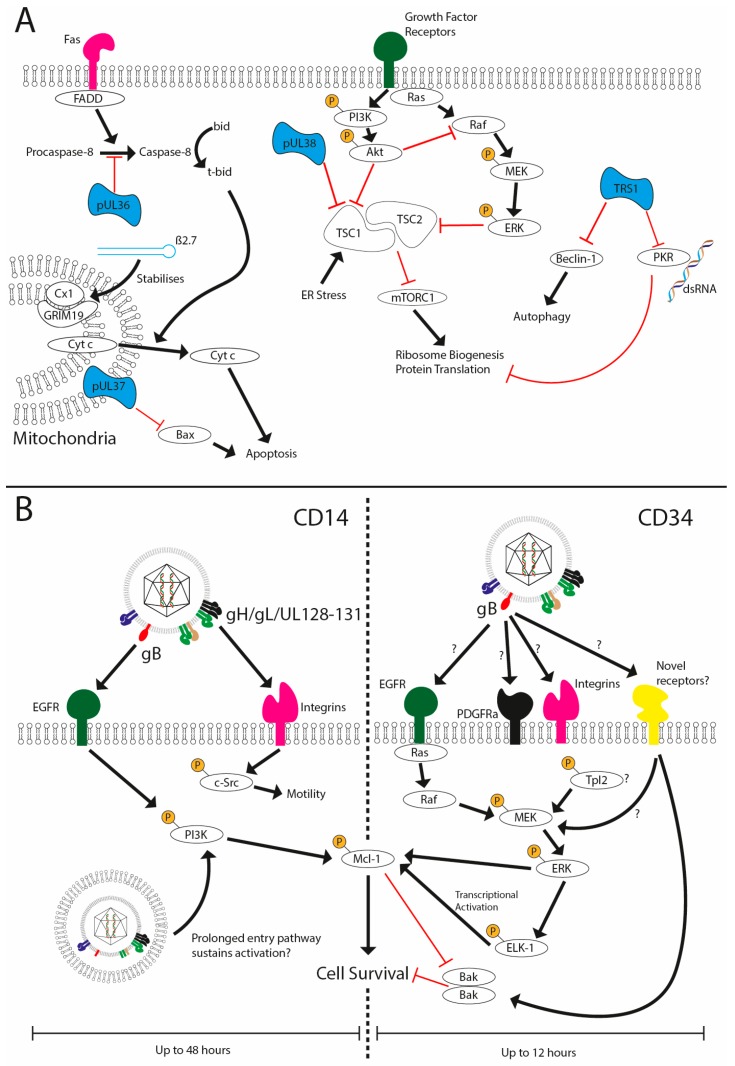
HCMV prevents cell death during lytic and latent infection. (**A**) HCMV has been shown to encode multiple viral inhibitors (represented in blue) of cell death pathways expressed during lytic replication to prevent extrinsic (UL36), intrinsic (UL37x1, Beta 2.7) and stress-induced cell death and autophagy (UL38 and TRS1). (**B**) HCMV infection of CD14 or CD34+ cells is non-lytic and, furthermore, results in the establishment of a latent infection in CD34+ cells. Viral glycoprotein complexes bind to the cell surface receptors to initiate signalling cascades that support survival. In CD14+ cells concomitant activation of EGFR and integrins via gB and the pentameric complex, respectively, promote sustained activated of PI3K activity and src signalling. The traffic of the HCMV capsid to the nucleus can take upwards of three days, which may underpin sustained PI3K activity and downstream effects on the expression of anti-apoptotic MCL-1. In contrast, the activation of ERK-MAPK via an unknown receptor in response to gB is responsible for survival. This activation is transient (1–8 hpi) but triggers a rapid upregulation of MCL-1 protein expression via the activation of the ELK-1 transcription factor. The net effect is to generate an anti-apoptotic state that negates the concomitant activation of death pathways that occurs during viral entry.

One of the major challenges facing HCMV in its attempt to establish latency is a requirement to evade host immune responses following infection in the absence of expression of the vast array of cell modulators encoded within its genome. One such host response is the activation of apoptosis as a mechanism to remove a replication niche for the pathogen. For instance, during lytic infection, the expression of proteins such as UL36, UL37, UL38 and TRS1 as well as lncRNAs such as β2.7 restrict cell death pathways (Figure 2A), allowing the virus to complete its replication cycle [80,81,82,83,84,85]. However, does this present an issue during the establishment and maintenance of latency? Many of these gene products are not expressed during the early stages of infection of non-permissive cells, so how does the virus keep infected cells alive? 

**Figure 3:**
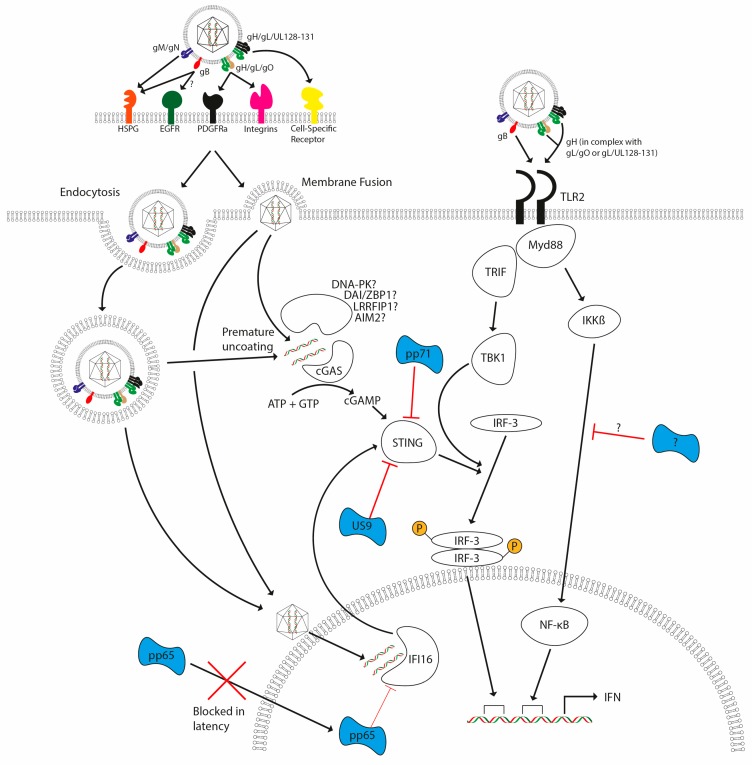
HCMV detection by the host and subsequent evasion strategies. HCMV lytic infection (either via endocytosis or membrane fusion) triggers TLR- and DNA-sensing pathways upon infection, with these pathways leading to the induction of interferon (and other antiviral cytokines). TLR2 activation by HCMV glycoproteins gB and gH lead to activation of TRIF and MyD88 pathways. DNA-sensing pathways involving cGAS and possibly other documented sensors signal via STING and TBK-1, leading to the activation of IRF3. TLR signalling activates NF-κB and IRF3 via MyD88 and TRIF, respectively. Failure to antagonize these responses leads to activation of interferon responses. Antagonists are illustrated in blue and are delivered with the virion (pp65, pp71) as well as being encoded during infection (pp71 and US9) to inhibit host detection systems. A direct antagonist of IKK activation via TLRs has not yet been reported, but it is clear that multiple steps of the pathways are antagonised by CMV.

A simple solution to the conundrum would be that viral infection of non-permissive cells does not activate cell death pathways at all. However, studies with multiple viruses imply that this is not the case. This is exemplified in the context of HCMV, CD14+ monocytes, CD34+ cells and the THP1 cell line (Figure 2B). It has been demonstrated that infection upregulates host cell survival signals and that blockade of this, or depletion of the survival signal itself, promotes cell death when challenged with the virus [86,87,88]. The key aspect was that the virus upregulated a cell-encoded anti-apoptotic protein that is a pivotal regulator of myeloid cell viability—myeloid cell leukaemia-1 (MCL-1) protein—to counter the inevitable host activation of cell death pathways [86]. A subsequent study demonstrated that infection of CD34+ cells with HCMV results in a transient activation of the pro-apoptotic protein Bak [88]. In cells where the survival signal is blocked, the activation of Bak is prolonged, likely contributing to cell death. The HCMV-induced survival effect was highly dependent on ERK-MAPK signalling, which was activated in a gB-dependent manner [86,88]. However, the precise identity of the receptor activated by gB in CD34+ cells remains to be determined, although a putative role for the DLD domain of gB [86] may argue for a key role for integrins [53]. It is likely that the identification of the receptor(s) being used by HCMV will also shed light on the upstream pathways activating the MEK-ERK pathway required for survival (Figure 3).

A similar model applies to the infection of CD14+ cells, whereby MCL-1 upregulation was also demonstrated to be required for the survival of CD14+ monocytes following HCMV infection. However, differences are evident. Firstly, the effect in monocytes was PI3K-dependent [87]. Secondly, MCL-1-mediated protection from apoptosis is a relatively transient event in CD34+ cells, with latently infected CD34+ cells no longer protected from cisplatin A-induced apoptosis 12 h post-infection [86]. However, unlike in CD34+ cells, MCL-1 levels remain elevated in CD14+ monocytes until at least 48 h post infection (hpi) [87], a long time after the initial binding events responsible for triggering its upregulation. Reasons for these differences are unclear, but the extended upregulation of MCL-1 and prolonged activation of PI3K may be linked to the unusual route that HCMV utilises during the infection of monocytes [78]. Additionally, activation of Akt by PI3K and the triggering of ERK-MAPK signalling lead to inhibition of Tuberous Sclerosis Complex 2 (TSC2) [89,90]. TSC1/2 inhibits mammalian target of rapamycin complex 1 (mTORC1) function, thereby inhibiting translation of proteins and ribosome biogenesis [91,92]. This signalling activity may compensate for the inability to express pUL38, which inhibits TSC1 function during lytic infection [93].

It is highly likely that the ability of HCMV to act as a poly-ligand through engagement of multiple receptors is critical for the survival response. Essentially, survival is the outcome of the activation of multiple signalling cascades working in concert to promote viability and only when these pathways are activated at the same time is protection observed.

### 3.2. Innate Immune Mechanisms for Detection of CMV

Underpinning the cell-intrinsic innate immune signalling apparatus that detects different components of virus particles is the activation of the production of interferon (IFN) and antiviral cytokines. This system consists of a series of pattern recognition receptors (PRRs) that detect various pathogen-associated molecular patterns (PAMPs). The role of the innate immune response to a viral infection is dual—to initiate a primitive antiviral state in the infected and neighbouring cells, and to help recruit cells of the adaptive immune system to commence a more specific response.

It is likely that viral glycoproteins such as gB and gH are detected at the cell surface, whereas other components such as viral nucleic acid can be detected at both the cell surface and intracellularly (Figure 3). Toll-like receptors (TLRs) are a series of membrane-associated PRRs that have been shown to detect a variety of PAMPs involved in HCMV infection, such as TLR9 detecting hypomethylated CpG DNA motifs, and TLR2 recognition of gB and gH at the cell surface [94]. Activation of TLRs leads to a downstream signalling cascade involving adapter molecules MyD88 and TRIF, culminating in the activation of the transcription factors NF-κB and IRF3 respectively. Monocyte activation in response to HCMV has been shown to be MyD88-dependent, suggesting a role for TLR sensing in response to HCMV in monocytes [95]. Whilst the induction of cytokines such as IFN has been shown to be partially dependent upon TLR signalling, it has been demonstrated that infection of mice with murine cytomegalovirus (MCMV) leads to a biphasic production of IFN [96], with the initial phase of IFN being, in fact, TLR-independent [97].

Beyond cell-surface receptors for viral components, the other major route for detection of viral infection involves the detection of viral DNA after the viral nucleic acid has become exposed to the cell. Mechanisms of detection of viral DNA have come under intense scrutiny following initial reports that demonstrated activation of IRF3 in response to viral, bacterial and synthetic DNA [98].

Multiple receptors, including cGAS, IFI16, DNA-PK, DAI, LRRFIP-1 and RNA Polymerase III have been shown to recognise DNA, activating innate signalling that leads to the transcription of interferon and other antiviral cytokines [99]. Following detection by DNA sensors, the downstream signalling pathway utilises long-known kinase molecules such as TBK-1, but there are upstream proteins specific to DNA. The number of DNA sensors described highlights a complexity to DNA sensing that is not yet fully appreciated. Various DNA viruses occupy separate niches that may require sensing by different molecules. For example, vaccinia virus is a cytoplasmic DNA virus that has been shown to be detected by DNA-PK [100] and the virus encodes an inhibitor of this molecule [101]. However, whilst DNA-PK has been shown to be present in many cell types, it is not expressed in macrophages, for example, which suggests that another DNA sensor is active in these cells. Whilst much attention has been focused on DNA sensing using synthetic DNA, the sheer variety of sensors is indicative either of redundancy in the system or, perhaps more likely, of the fact that different sensors could play cell or intracellular compartment specific roles in response to pathogens.

A key player on which the DNA sensing pathways converge is the adapter molecule STING. As such, STING has been shown to be critical for the activation of IRF3 in response to DNA via TBK1 during HCMV infection [102]. Similarly, STING was identified to be essential for the initial burst of interferon in response to HCMV infection in primary human endothelial cells [103], suggesting a role for intracellular DNA sensing in the initial response to HCMV. Further evidence of the importance of STING to the sensing of HCMV DNA comes from recent studies that have demonstrated that HCMV encodes multiple inhibitors of STING (US9 [104] and pp71 [105]). Tegument protein pp71, which is delivered with the virion upon entry, targets STING for degradation. It is likely that this is dependent on the LxCxD motif present in pp71 that is required for its interaction with retinoblastoma protein [106], since work from the Stetson laboratory has shown that the related LxCxE motif in SV40 T antigen is required for directed degradation of STING [107]. Indeed, the study of pp71 that identified the domain responsible for targeting STING contains the LxCxD motif [105].

The mechanism of detection of HCMV DNA upstream of STING is not yet fully established and could possibly involve multiple detectors acting in cell-type-specific ways. It has been reported that DAI plays a role in the detection of HCMV DNA in fibroblasts [108], despite the importance of DAI having been doubted given the absence of any deficit of IFN production in response to DNA in DAI-/- MEFs and mice [108,109]. Furthermore, IFI-16 has been suggested to play a role in detection of HCMV detection as disruption of IFI-16 with shRNA resulted in reduced expression of cytokines and reduced activation of IRF-3 following infection with HCMV [93]. The same study also found a role for the tegument protein pp65 in disrupting IFI-16-mediated signalling, suggesting an important role for IFI-16 in the detection of HCMV DNA. Thus two tegument proteins (pp65 and pp71) delivered with the virion are targeting key components of the DNA sensing pathway. Indeed, it is possible that the delivery of the antagonists with the virion becomes pertinent during the establishment of latency—a time where very limited gene expression is observed. Finally, a further study also showed a role for cyclic GMP-AMP synthase (cGAS) in innate immune control of HCMV since endothelial cells lacking cGAS produced lower levels of interferon compared with control cells. This finding was replicated with STING and TBK-1 knockdown, further emphasising the importance of IRF-3-mediated signalling in the initial response to HCMV [103].

Beyond IRF-3 signalling, another PRR termed AIM2 has been shown to recognise DNA and initiate the formation of an inflammasome that leads to the elaboration of IL-1β and IL18-independent of IRF-3 signalling. It is not fully understood if inflammasomes play an important role in the control of HCMV infection. Inflammasomes are multimolecular bodies that convert pro-IL-1β and pro-IL-18 into their active forms, IL-1β and IL-18. AIM2 has been well characterised as a DNA sensor present in macrophages; however, the exact role it plays in HCMV has yet to be established. It has been noted that monocyte activation in response to HCMV is independent of the inflammasome adapter protein ASC [95], perhaps suggesting that DNA sensing via AIM2 is either powerfully inhibited by the virus, or non-essential.

Whilst cytokines and chemokines undoubtedly primarily function to mount an immune response against HCMV, the cytokines involved have also been shown to have an effect on the latency of herpesviruses. HSV-1 latency in vitro has been shown to be enhanced by the production of type I IFNs [110], and several other herpesvirus latency states are shown to be IFN-sensitive. For example, systemic administration of type I IFN to mice has been shown to prevent MCMV reactivation [111]—presumably via the known inhibitory activity of IFN against viral IE gene expression [112]. The authors postulated that IFN treatment enhanced the activity of PML—an interferon-stimulated gene that is a well-established inhibitor of lytic gene expression of multiple herpes viruses [113]. However, no direct evidence for a role for PML was shown and it is interesting to note that in HCMV infection of non-permissive THP1 cells the presence or absence of PML has been reported to have no impact on the establishment of latent infections [114].

What all these studies of HCMV may reveal is that the nature of the innate immune response to HCMV differs depending on which cell types are studied and the entry mechanism used. The influence of the differing responses of cell types and how this might impact on the progression of the infection of HCMV warrants further investigation. One potential conceptual challenge is the question of whether the host or the virus benefits the most from an IFN-based response against the virus. Whilst IFN may be important for keeping herpesviruses at bay, if it contributes to or even enhances the establishment of latency then, on an epidemiological level, this is potentially advantageous. An obvious benefit from lifelong cycles of latency and reactivation is the increased opportunity for transmission to new hosts.

The lack of HCMV virus production during latency suggests that many of the pathways used to detect viruses may not be activated during latency. However, whether HCMV DNA is a potential long-term PAMP during latency is unclear. This question is linked to the broader issue of how the viral DNA is distinguished from cellular DNA by the DNA sensing machinery. In the case of vaccinia, a cytoplasmic DNA virus, the cellular location of the DNA may be the defining feature. However, HCMV DNA exists in a circularised form in the nucleus during latency [41], and, furthermore, the HCMV episomes are extensively chromatinised [33]. It is possible that this renders it indistinguishable to DNA sensors from cellular DNA and therefore not detected at all during latency. This would assume that sequence information is not important for recognition by DNA sensors. For example, if viral genomes contain an abundance of repetitive sequences favoured by DNA sensors, it may be a mechanism for the sensors to selectively identify foreign DNA. Certainly, sequence-specific recognition of foreign DNA could be important. A recent study of HIV revealed that the presence of CG di-nucleotides is markedly reduced in the genome—an adaptation that potentially protects it from recognition by host zinc finger antiviral proteins with potent antiviral activity [115].

The sensors playing a role in sensing of HCMV have yet to be fully tested. Whilst a role for HCMV was suggested by the existence of an inhibitor of IFI-16 in pp65, more recent studies have demonstrated that genetic knockout of AIM2-like molecules (including IFI-16) using CRISPR have shown that IFI-16 is dispensable for the induction of IFN in response to HCMV, conflicting with data generated using an siRNA approach in human fibroblasts [116]. The presence of the HCMV-encoded inhibitor pp65 has been accounted for by using a virus deficient in this molecule in the study and IFI-16 remained non-essential. Clearly, there is a complexity to HCMV DNA sensing that has yet to be fully understood and may again indicate that cell-type-specific responses are key.

## 4. Conclusions and Future Perspectives

The modulation of the cellular environment is initiated at the point of entry via the activation of signalling pathways. Furthermore, within the virion are several tegumental phosphoproteins such as pp65 and pp71 that are rapidly delivered to the nucleus upon infection of differentiated cells such as fibroblasts. Here they can counteract antiviral responses dictated by interferon-induced PML bodies and components of the DNA sensing pathway. For instance, pp71 targets Daxx [117,118], leading to Daxx SUMOylation [119] and its destruction by the proteasome [118]. This removes a cellular repressor of viral IE expression, leading to the production of IE72. IE72 then targets PML bodies for disruption and allows the virus to overcome this potent host defence [120]. The failure of pp71 to translocate to the nucleus of infected CD34+ cells—and, consequently, a failure to degrade Daxx—has been postulated as an event contributing to the establishment of latency. Heterokaryon experiments using NTERA-2 teratoma cells [121], an experimental model of quiescent infection, fused to fibroblasts demonstrated that permissive fibroblasts possess a factor that supports the trafficking of both pp71 and pp65 to the nucleus rather than undifferentiated cells encoding a factor that blocks translocation of these proteins. Changes to trafficking may also explain the transient permissiveness of LPS-stimulated monocytes, as discussed earlier, as LPS may induce a factor required for tegument delivery to the nucleus, allowing for pp71 localisation and antagonism of PML body formation, but this is not sufficient to drive the virus through its lifecycle in an otherwise non-permissive cell.

The hypothesis that the activity of PML bodies may be beneficial for the establishment of latency is an intriguing possibility. However, contrasting data have demonstrated that knockdown of individual PML body components (PML, Daxx and sp100) [114,122] in T2 or THP-1 cells had no effect on the number of cells expressing IE2 following infection, suggesting that removal of individual components had not led to the production of lytic gene transcripts. There was a difference in the number of cells expressing IE2 following differentiation of infected THP-1 cells, with knockdown of each component leading to increased numbers of IE2 expressing cells. This suggests that PML bodies may affect the efficiency of reactivation but not the establishment of a quiescent infection. However, it was not shown that the number of quiescent genomes was similar between the different knockout cell lines, raising an alternative possibility that PML body component knockdown allowed more genomes to establish a latent infection without an increase in the number of cells expressing lytic genes.

Finally, it may be worth considering the possibility that the differential localisation of tegument proteins during infection of non-permissive cells is beneficial during latency for other reasons. An abundance of pp71 in the cytoplasm may be important to prevent the activity of STING and consequential DNA sensing of the incoming latent genomes. Alternatively, the occlusion of pp65 and pp71 from the nucleus may promote a transient antiviral state that is interferon-dependent based on a failure to prevent sensing and IFN signalling. Studies in HSV have implicated that type I interferons may support the establishment of latency and thus a transient induction may benefit HCMV. In some ways, it is not unsurprising that HCMV utilises aspects of the antiviral IFN response. Multiple studies of lytic infection have suggested that IFN-stimulated genes such as tetherin [123] and IFITM3 [124] could play a positive role in HCMV infection, although it is interesting to note a recent observation that long-term latent infection of CD34+ cells with HCMV results in a downregulation of multiple HIV-associated restriction factors [125]. The biological advantage of their downregulation to latent HCMV remains unclear, but it does suggest that restriction factors may be detrimental during latent carriage of HCMV genomes.

Whilst the precise impact of cellular restriction factors on the ability of HCMV to establish latency is still subject to investigation, perhaps in vitro models have the potential to generate a misleading picture of HCMV latency in vivo. For example, it is possible that interferons and pathogen detection have no role to play in the establishment of latency in early progenitor cells. A recent and compelling study of stem cells indicated that these cells, which are highly resistant to viral infection, encode high levels of endogenous interferon-stimulated genes (ISGs) [126]. Crucially, their expression was independent of type I IFNs. Consequently, our attempts to model early events during HCMV latent infection in vitro using more differentiated cells (and, specifically, attempts to analyse the effects of the interferon-dependent innate immune responses) may not fully reflect the event in vivo, although, inadvertently, in both instances may reflect a role for ISGs in the establishment of latency. Key to determining whether this is a factor is the identification of the specific progenitor cell type infected by HCMV. Lineage commitment was associated with acquisition of interferon sensitivity and thus HCMV may establish latency in lineage-committed cells rather than a truly pluripotent stem cell.

In conclusion, the outcome of infection is a concert of effects triggered by the pathogen and the host. Understanding the triggers of the host responses and the subsequent mechanisms pathogens use to subvert them can give us insight not only into the host–pathogen interaction but can also shed new light on the complex function of the underlying components that regulate these multifaceted host responses.
